# Indoleamine 2,3-dioxygenase 1 signaling orchestrates immune tolerance in *Echinococcus multilocularis*-infected mice

**DOI:** 10.3389/fimmu.2022.1032280

**Published:** 2022-11-11

**Authors:** Ru Meng, Yong Fu, Yaogang Zhang, Yalin Mou, Gongguan Liu, Haining Fan

**Affiliations:** ^1^ Research Center for High Altitude Medicine, Key Laboratory of High Altitude Medicine (Ministry of Education), Key Laboratory of Application and Foundation for High Altitude Medicine Research in Qinghai Province (Qinghai-Utah Joint Research Key Lab for High Altitude Medicine), The Research Key Laboratory for Echinococcosis of Qinghai Province, Qinghai University, Xining, China; ^2^ Academician Zhang Yong Innovation Center, Xining Animal Disease Control Center, Xining, China; ^3^ Qinghai Academy of Animal Sciences and Veterinary Medicine, Qinghai University, Xining, China; ^4^ Qinghai University Affiliated Hospital, Qinghai University, Xining, China; ^5^ Key Laboratory of Animal Diseases Diagnostic and Immunology, Ministry of Agriculture, MOE Joint International Research Laboratory of Animal Health and Food Safety, College of Veterinary Medicine, Nanjing Agricultural University, Nanjing, China

**Keywords:** alveolar echinococcosis, *Echinococcus multilocularis*, dendritic cells, indoleamine 2,3-dioxygenase 1, immunosuppression

## Abstract

The cestode *Echinococcus multilocularis* larva infection causes lethal zoonotic alveolar echinococcosis (AE), a disease posing a great threat to the public health worldwide. This persistent hepatic tumor-like disease in AE patients has been largely attributed to aberrant T cell responses, of which Th1 responses are impeded, whilst Th2 and regulatory T cell responses are elevated, creating an immune tolerogenic microenvironment in the liver. However, the immune tolerance mechanisms are not fully understood. Dendritic cells (DCs) are key cellular components in facilitating immune tolerance in chronic diseases, including AE. Here, we demonstrate that indoleamine 2,3-dioxygenase 1-deficient (IDO1^-/-^) mice display less severe AE as compared to wild-type (WT) mice during the infection. Mechanistically, IDO1 prevents optimal T cells responses by programming DCs into a tolerogenic state. Specifically, IDO1 prevents the maturation and migration potential of DCs, as shown by the significantly enhanced expression of the antigen-presenting molecule (MHC II), costimulatory molecules (CD80 and CD86), and chemokine receptors (CXCR4 and CCR7) in infected IDO1^-/-^ mice as compared to infected wild-type mice. More importantly, the tolerogenic phenotype of DCs is partly reverted in IDO1^-/-^ mice, as indicated by enhanced activation, proliferation, and differentiation of both CD4^+^ and CD8^+^ - T cells upon infection with *Echinococcus multilocularis*, in comparison with WT mice. Interestingly, in absence of IDO1, CD4^+^ T cells are prone to differentiate to effector memory cells (CD44^+^CD62L^-^); in contrast, CD8^+^ T cells are highly biased to the central memory phenotype (CD44^+^CD62L^+^). Overall, these data are the first to demonstrate the essential role of IDO1 signaling in inducing immunosuppression in mice infected with *Echinococcus multilocularis*.

## Introduction

Alveolar echinococcosis (AE) is a lethal zoonotic disease caused by infection with the larva of the cestode *Echinococcus multilocularis* (*E. multilocularis*) (1). Humans are occasional intermediate hosts, and acquire the infection through ingestion of food and water contaminated with eggs expelled with the feces of canid species (2). The larval cells predominantly reside in the liver, progressively invade neighboring tissues and organs, and eventually lead to a nearly 90% mortality rate if remaining untreated (3, 4). AE is becoming an emerging/re-emerging disease worldwide, mainly attributable to the lack of vaccines and active control measures (2, 5). Of note, *E. multilocularis* exploits efficient immune evasion mechanisms to establish persistent infection (6). However, the mechanism by which *E. multilocularis* induces host immune tolerance, is still unclear. Therefore, it is vitally important to unveil the immune mechanism of AE and explore effective immunological prevention measures.

Dendritic cells (DCs), the most professional antigen-presenting cells, are the critical mediators in the activation of cellular immune responses as well as tolerance induction (7). During *E. multilocularis* infection, DCs display phenotypical and functional alterations favoring disorders of the anti-parasite immunity, of which Th2- and Treg- responses are enhanced to promote the tumor-like progression of the parasitic metacestodes (8–10). In contrast, IFN-γ dependent effector mechanisms, including Th1 responses, abrogate parasite survival, proliferation and maturation (8, 11, 12). Although the general importance of DCs in the host-parasite interaction has been largely appreciated (13), immunomodulatory molecules involved in DCs functions are still not well characterized in *E. multilocularis* infection.

Indoleamine 2,3-dioxygenase (IDO) is an essential enzyme catalyzing the initial and rate-limiting step in the catabolism of tryptophan and serotonin in the kynurenine pathway (14, 15). The resultant catabolite, namely kynurenine, is further degraded into kynurenic acid and other catabolites by enzymes downstream of IDO. The IDO-initiated metabolic changes can exert potent immunomodulatory effects. For instance, resting regulatory CD4^+^ T cells (Tregs) become activated upon sensing the degradation of tryptophan, resulting elevated tolerogenic molecular effectors such as IL-10 and TGF-ß (16, 17). Three enzymes with IDO activity exist, namely indoleamine 2,3-dioxygenase 1 (IDO1), indoleamine 2,3-dioxygenase 2 (IDO2) and tryptophan 2,3-dioxygenase (TDO) (18, 19). In particular, IDO1, endowed with non-enzymatic signaling activity in DCs, promotes immune tolerance by altering DCs functions (20–23). Previous studies have revealed that elevated IDO1 expression on DCs is of particular significance due to the tolerogenic effects on T cell responses (24–27). Recent reports suggest that IDO1 activity converts mature DCs into a tolerogenic phenotype that suppresses effector T cell function and promotes tolerance (28). Moreover, activation of IDO1signaling reduces the T cell dependent immunotherapeutic efficacy in several types of cancer diseases, including the hepatocellular carcinoma (29–32). Emerging evidence has demonstrated that DCs are involved in immune tolerance during *E. multilocularis* infection (33–35). However, the effects of IDO1 on DCs functions during *E. multilocularis* infection have not been elucidated, especially with regard to how the IDO1 signaling mediates potent immune tolerance effects *in vivo*.

Herein, taking advantage of magnetic resonance imaging and flow cytometry, we found that infection of *E. multilocularis* induced enhanced expression of IDO1 on DCs of wild-type (WT) mice, whereas, mice in absence of IDO1 exhibited reduced progression of metacestode tissue and decreased parasite loads in the mice. Mechanistically, activation of IDO1 signaling prevented the activation, proliferation, and differentiation of T helper subtype 1 cells (Th1 cells) and cytotoxic T lymphocytes cells (CTLs), which was attributed to the reduced maturation of DCs in terms of the expression of costimulatory molecules and chemokine receptors. We conclude that IDO1 promotes immunosuppression and alveolar echinococcosis progression.

## Materials and methods

### Mice, parasites and infection

Eight- to ten-week-old C57BL/6 mice and IDO1-deficient (IDO1^-/-^) C57BL/6 mice were purchased from the Jiangsu GemPharmatech Co. Ltd., and were bred in a specific pathogen-free environment with a 12-h light/dark cycle supplemented with rodent chow and water ad libitum.

*E. multilocularis* protoscoleces (PSCs) were obtained from intraperitoneal lesions maintained in BALB/c mice under aseptic conditions, followed by three rounds of clean-up with phosphate buffered saline (PBS, pH=7.2, containing 1000 mg/mL penicillin and 1000 U/mL streptomycin) (36). PSCs with over 95% vitality determined by eosin exclusion were counted using hemocytometer (37), and 1000 PSCs were injected intraperitoneally per mice as previously described (38). To determine the parasitic burden using wet-weighing method, mice were sacrificed in month 1, 2 or 3 post-infection, and parasitic tissues were dissected from the liver and the peritoneal cavity.

### Antibodies

Alexa Fluor^®^ 647 anti-mouse IDO1 (2E2/IDO1), PE anti-mouse CD11c (N418), PerCP/Cyanine5.5 anti-mouse/human CD11b (M1/70), Brilliant Violet 650™ anti-mouse CD80 (16-10A1), APC anti-mouse CD86 (GL-1), FITC anti-mouse I-A/I-E (M5/114.15.2), PerCP/Cyanine5.5 anti-mouse CD45 (30-F11), APC/Fire™ 750-anti-mouse CD3 (17A2), FITC anti-mouse CD4 (RM4-5), Brilliant Violet 650™ anti-mouse CD8a (53-6.7), APC anti-mouse CD62L (MEL-14), PE/Dazzle™594 anti-mouse/human CD44 (IM7), PE/Dazzle™594-anti-mouse CD184 (CXCR4) (L276F12), Brilliant Violet 421™ anti-mouse CD197 (CCR7) (4B12), PE anti-human/mouse Granzyme B (QA16A02), Brilliant Violet 421™ anti-mouse IFN-γ (XMG1.2), Brilliant Violet 421™ anti-mouse Ki-67 (16A8) were purchased from Biolegend, USA.

### Purification of splenocytes

Splenocytes were purified as described previously (39). In brief, the spleen was removed, cut into small pieces with surgical scissors, and then mechanically dispersed by using a sterile syringe plunger to force through a 70-μm cell strainer in 50 mL RPMI 1640 medium containing 5% fetal calf serum (FCS). The cell suspension was centrifuged at 500 g for 5 min at 4°C, then the pellet was resuspended in 1 mL ACK buffer (erythrocyte lysing buffer) and incubated at room temperature for 3-5 min, followed by addition of 14 mL cold RPMI 1640 medium. After centrifugation at 500 g for 5 min at 4°C, cells were resuspended in cold RPMI 1640 medium containing 5% FCS.

### Flow cytometry

Splenocytes were incubated with TruStain FcX™ PLUS anti-mouse CD16/CD32 (Biolegend; clone: S17011E) for 10 min at 4°C, and then stained with mAbs specific for various cell surface markers. To evaluate the production of IFN-γ and Granzyme B, splenocytes were diluted to 4 × 10^6^ cells/mL and cultured (500 μL/well) in a 24-well plate containing Cell Activation Cocktail (with Brefeldin A; Biolegend) for 6 h. Cells were then harvested and washed twice in cell staining buffer prior to surface marker staining as described above. Cells were then fixed and permeabilized using Intracellular Fixation Buffer & Intracellular Staining Permeabilization Wash Buffer (Biolegend). Intracellular staining was then performed using mAbs specific for IFN-γ and granzyme B, respectively. For Ki-67 staining, freshly isolated splenocytes were stained following the above-mentioned surface and intracellular staining procedures, with no *in vitro* stimulation. Samples were resuspended in cell staining buffer, tested with BD FACSCelesta flow cytometer, and analyzed using FlowJo software (BD, USA).

### Magnetic resonance imaging (MRI)

MRI scanning was performed using a small-animal MRI Facility (Bruker PharmaScan 70/16 US, Germany) with the Paravision 360 software platform. The scanning coil is mouse body coilRF RES 300 1H 075/040 QSN TR. The mice were anesthetized by inhalation with 2% ~ 3% isoflurane (RWD, Shenzhen) during scanning. The body temperature of mice was kept constant throughout the experiment using a thermoregulated water circulation system, and the oxygenation level of the mice was monitored by a pulse oximeter. The scan protocols for axial images were performed using the T2_TurboRARE as described previously (40). In brief, echo time (TE) = 25 ms, repetition time (TR) = 2100 ms, slice thickness = 1.0 mm, field of view (FOV) = 35 mm×30 mm, scanning time = 17 min 42s; T1_FLASH_flc: TE = 3 ms, TR = 300 ms, flip angle = 90°, slice thickness = 1.0 mm, FOV = 35 mm×30 mm, scanning time = 5min 45s. The maximum diameter of metacestodes was quantified and analyzed using T1 Imaging and T2 imaging of Sante MRI Viewer V3.0.

### Mixed lymphocyte reaction (MLR)

DCs were purified from the spleens of the *E. multilocularis* infected IDO1^-/-^ and wild-type (WT) mice using CD11c Microbeads UltraPure (Miltenyi Biotech). 5×10^5^ DCs per well of each genotype were seeded into 24-well plate with or without the stimulation of LPS (Sigma). 24 h later, DCs were further treated with mitomycin C for 30 min, and then washed with sterile PBS for downstream experiments. Splenic CD4^+^ T cells from naïve BALB/c mice (purified with CD4 Microbeads, Miltenyi Biotech) were labeled with CFSE (Thermo Fisher Scientific) according to the manufacture’s instruction, and co-cultivated with the above mentioned DCs with the ratio of DCs:T cells at 1:4. Wells only containing CFSE stained naïve CD4^+^ T cells were prepared as negative controls. On day 4 of the co-cultivation, cells were collected, and the CFSE dilution was assessed using flow cytometry to determine the T cell proliferation.

### Statistical analysis

Data were represented as the mean ± SEM. Significance of differences was determined by 2-tailed unpaired *t*-test or ANOVA using the GraphPad Prism 9.0 software. *p* values<0.05 were considered statistically significant.

## Results

### *E. multilocularis* infection induces enhanced expression of IDO1 on DCs

**Figure 1:**
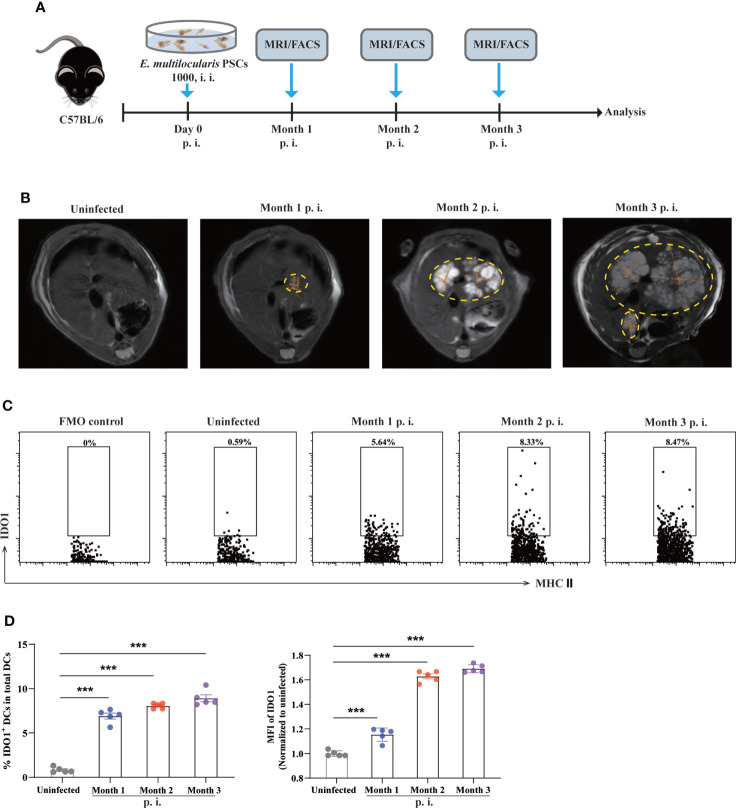
Expression of IDO1 on splenic DCs in murine *E multilocularis* infection model. **(A)** Schematic representation of the experimental setup. **(B)** Representative MRI images of metacestode tissue in the liver from *E multilocularis*–infected mice, and metacestode tissues were manually encircled with dashed yellow lines. **(C)** Representative flow cytometry plots of IDO1 on splenic CD11c^+^MHC II^+^DCs in infected and non-infected C57BL/6 mice (n = 5/group). **(D)** The frequency of IDO1 expressing splenic CD11c^+^MHC II^+^DCs derived from infected mice and non-infected controls (n = 5/group). Splenic CD45^+^MHC II^+^CD11c^+^ cells were gated. Data are pooled from 2 independent experiments, and presented as the mean ± SEM. ***, *p<*0.001. PSCs, protoscoleces; p.i., postinfection; i.i., intraperitoneal injection; MRI, magnetic resonance imaging; MFI, median fluorescence intensity; FMO, full minus one staining control.

To investigate the expression level of IDO1 on DCs during *E. multilocularis* infection, we employed an AE mouse model in which C57BL/6 mice were intraperitoneally injected with *E. multilocularis* protoscoleces (PSCs). (**Figure 1A**). Taking advantage of MRI scanning, tumor-like metacestode tissues were observed in the liver of infected mice, indicating successful establishment of murine AE model (**Figure 1B**). To determine the expression of IDO1, mice were sacrificed and splenocytes were analyzed using flow cytometry at multiple time points post infection with *E. multilocularis* (**Figure 1A**). We found that in uninfected mice, splenic CD11c^+^MHC II^+^ DCs barely expressed IDO1 (**Figures 1C, D**; **Supplementary Figure 1**). In contrast, splenic CD11c^+^MHC II^+^ DCs in *E. multilocularis* infected mice exhibited significantly higher frequency and intensity of IDO1 1-, 2- and 3- month post infection. (*p<*0.001; **Figures 1C, D**). These results suggest that metacestode tissue growth is positively correlated to the enhanced expression of IDO1 in DCs during *E. multilocularis*.

### IDO1 signaling promotes metacestode tissue progression in mice infected with *E. multilocularis*

**Figure 2:**
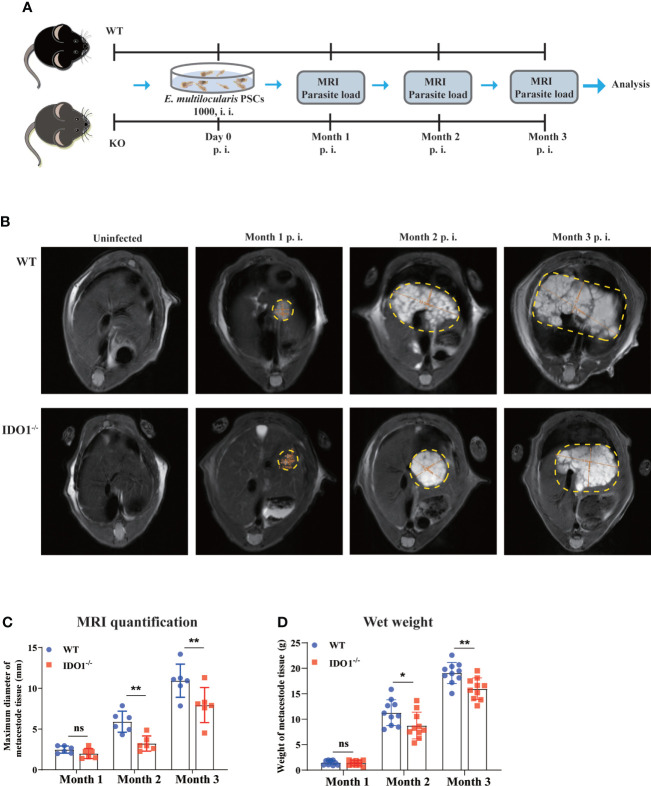
The effects of IDO1 on lesion area and parasite load in *E multilocularis* infected mice. **(A)** Schematic representation of the experimental setup. **(B)** Representative MRI images of metacestode tissue in the liver of IDO1^-/-^ and wild-type (WT) mice infected with *E multilocularis*, and metacestode tissues are manually encircled with dashed yellow lines. **(C)** The maximum diameter of metacestode tissues in IDO1^-/-^ and WT mice was quantified during MRI scanning using software in month 1, 2 and 3 post-infection with *E multilocularis* (n = 6/group). **(D)** The parasite load in IDO1^-/-^ and WT mice assessed by wet weight measurement at 1-, 2, and 3- month post-infection with *E multilocularis* (n = 10/group). Data are pooled from 2 independent experiments, and presented as the mean ± SEM. ns, not significant; *, *p<*0.05; **, *p<*0.01. PSCs, protoscoleces; p.i., post infection; i.i., injected intraperitoneally; MRI, magnetic resonance imaging.

Next, we infected IDO1^-/-^ and wild-type (WT) mice with *E. multilocularis* to assess whether IDO1 signaling affected the disease progression as illustrated in **Figure 2A**. MRI examination was used to depict the liver metacestode tissues of infected WT and IDO1^-/-^ mice, among which WT mice showed more extensive parasite lesions than IDO1^-/-^ mice in month 2 and 3 after infection (**Figures 2B, C**). To further evaluate the metacestode tissue progression, we measured the parasite load directly by weighing metacestode tissues. As shown in **Figure 2D**, IDO1^-/-^ mice exhibited a significantly lower parasite load compared to WT mice 2- and 3- month post infection (*p*<0.05 or *p*<0.01). These results demonstrate that IDO1 signaling plays an essential role in facilitating parasite growth in mice upon *E. multilocularis* infection.

### Deficiency of IDO1 signaling results in enhanced maturation of DCs in mice infected with *E. multilocularis*

**Figure 3:**
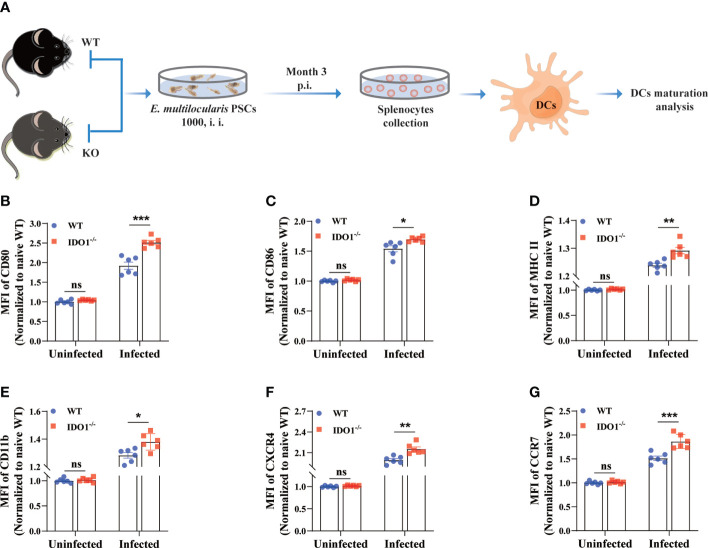
IDO1 signaling limits DCs maturation in mice infected with *E multilocularis*. **(A)** Schematic representation of the experimental setup. **(B–G)** Quantification of the relative intensity of CD80, CD86, MHCII, CD11b, CXCR4, and CCR7 within splenic DCs of the IDO1^-/-^ and WT mice at 3 months post-infection with *E multilocularis* (n = 6/group). CD45^+^MHC II^+^CD11c^+^ cells were gated. Data are pooled from 2 independent experiments, and presented as the mean ± SEM. ns, not significant; *, *p<*0.05; **, *p*< 0.01; ***, *p*< 0.001. PSCs, protoscoleces; p.i., post infection; i.i., injected intraperitoneally.

It has been reported that *E. multilocularis* infection prevents the maturation of DCs, as illustrated by the diminished expression of various costimulatory molecules and chemokine receptors (10, 41). To investigate whether IDO1 is involved in the maturation of DCs in mice infected with *E. multilocularis*, we next evaluated its effects on the expression of multiple phenotypic maturation markers, namely CD80, CD86, MHC II, CD11b, CXCR4, and CCR7, on DCs in infected IDO1^-/-^ and WT mice (**Figure 3A**). Strikingly, the intensity of all tested molecules on splenic DCs of infected IDO1^-/-^ mice was significantly higher than the counterparts on splenic DCs of infected WT mice (*p*<0.05, *p*<0.01 or <0.001, **Figures 3B–G**, **Supplementary Figure 2A-F**). Taken together, these results suggest that IDO1 signaling suppresses DCs maturation during infection with *E. multilocularis.*

### IDO1 deficiency mice exhibit increased T cells activation during *E. multilocularis* infection

**Figure 4:**
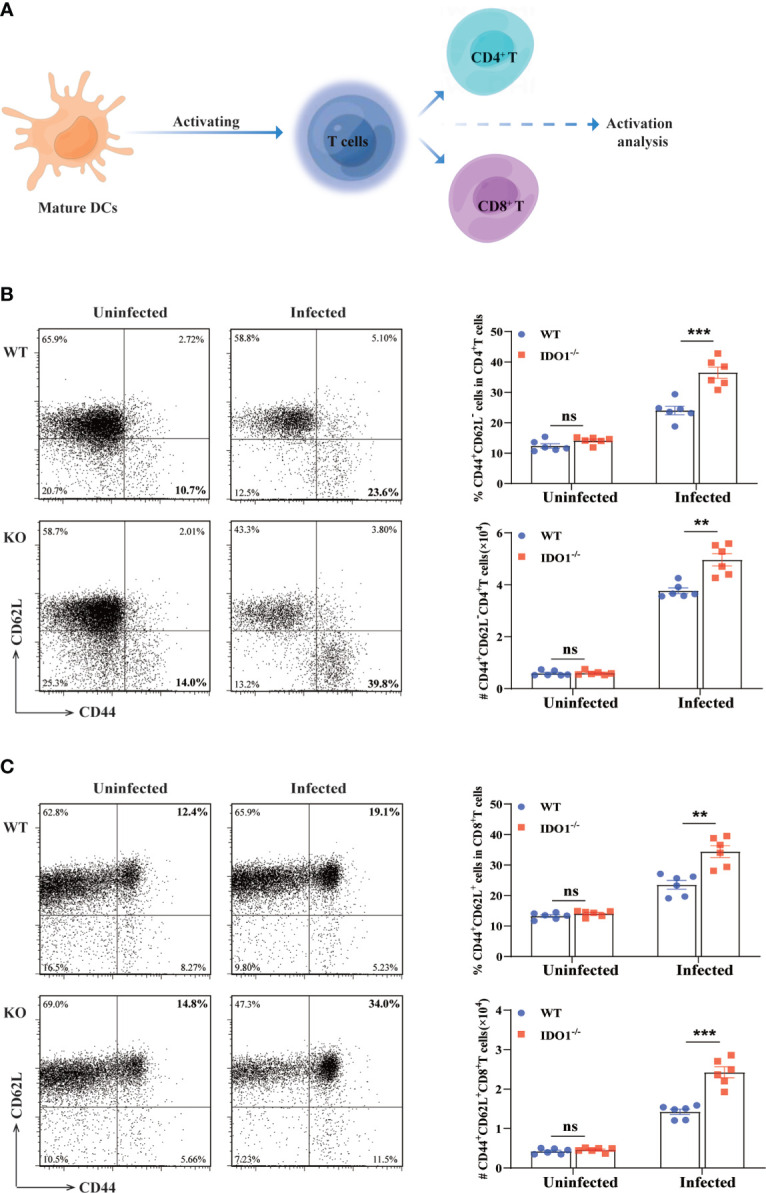
Enhanced activation of T cells in IDO1^-/-^mice infected with *E multilocularis*. **(A)** Conceptual schematic diagram. **(B, C)** Representative flow cytometry plots (left), the frequency (upper right), and the absolute number (lower right) of activated CD4^+^ T cells **(B)**, and CD8^+^ T cells **(C)** of IDO1^-/-^ and WT mice at 3 months post-infection with *E multilocularis* (n = 6/group). CD45^+^CD3^+^CD4^+^ and CD45^+^CD3^+^CD8^+^ cells were gated respectively for panel **(B, C)** Data are pooled from 2 independent experiments, and presented as the mean ± SEM. ns, not significant; **, *p*< 0.01; ***, *p*< 0.001.

Optimal T cell responses are essential to control the *E. multilocularis* infection in mice. To determine whether the phenotype of IDO^-/-^ DCs necessarily impacts the T cell response, we performed the Mixed Lymphocyte Reaction *in vitro*. Our results reveal that, in comparation with IDO1 sufficient DCs isolated from *E. multilocularis* infected WT mice, IDO1 deficient DCs from infected IDO1^-/-^ mice induce more robust T cell proliferative alloresponses (**Supplementary Figure 3**). CD44 and CD62L are common surface markers for assessment of major subsets of CD4^+^ T and CD8^+^ T cells in mice (42, 43). To further determine the effects of IDO1 on T cells activation during *E. multilocularis* inf ection, CD62L and CD44 were employed to assess naive (CD44^-^CD62L^+^), effector memory (CD44^+^CD62L^-^) and central memory (CD44^+^CD62L^+^) T-cell subsets in the spleens of IDO1^-/-^ and WT mice infected with *E. multilocularis* (**Figure 4A**). We found that the frequency and the absolute number of activated effector memory CD4^+^, but not CD8^+^, T cells (CD44^+^CD62L^-^) were significantly higher in IDO1^-/-^ mice infected with *E. multilocularis*, in contrast with infected WT mice (*p*<0.01 or <0.001, **Figure 4B**). Interestingly, the activated central memory CD8^+^ T cells (CD44^+^CD62L^+^) were significantly higher in infected IDO1^-/-^ mice when compared to that of infected WT mice (*p*<0.01 or <0.001, **Figure 4C**). In particular, the absolute number of activated central memory CD8^+^ T cells was increased by 2–3 folds in infected IDO1^-/-^mice (**Figure 4C**). Collectively, these data suggest that the IDO1 signaling prevents effector memory CD4^+^ T cells or central memory CD8^+^ T cells development in mice infected with *E. multilocularis.*

### Enhanced T cells proliferation in IDO1^-/-^ mice infected with *E. multilocularis*

**Figure 5:**
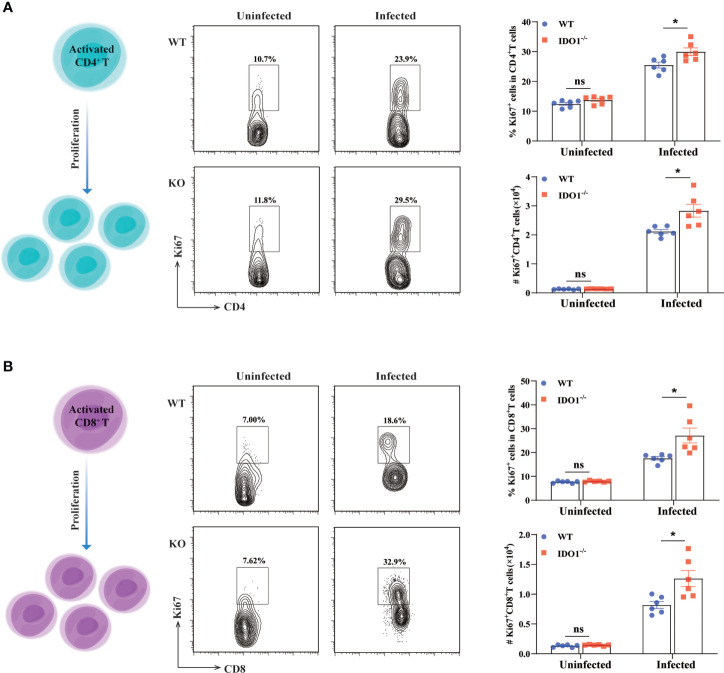
Enhanced proliferation of T cells in IDO1^-/-^mice infected with *E multilocularis.* **(A, B)** Conceptual schematic diagram (left), representative flow cytometry plots (middle), and the frequency (upper right) and the absolute number (lower right) of proliferating CD4^+^ T cells **(A)** and CD8^+^ T cells **(B)** of the IDO1^-/-^ and wild-type mice at 3 months post-infection with *E multilocularis* (n = 6/group). CD45^+^CD3^+^CD4^+^ and CD45^+^CD3^+^CD8^+^ cells were gated respectively for panel **(A, B)**. Data are pooled from 2 independent experiments, and presented as the mean ± SEM. ns, not significant; *, *p* < 0.05.

As shown above, deficiency of IDO1 signaling enhances the activation of T cells in mice infected with *E. multilocularis.* Next, we used Ki67, a nuclear protein expressed throughout the cell cycle in proliferating cells, to determine the proliferation of both CD4^+^- and CD8^+^- T cells during *E. multilocularis* infection. As shown in **Figures 5A, B** the frequency and the absolute number of Ki67 expressing splenic CD4^+^- and CD8^+^- T cells, were significantly higher than that in infected WT mice (*p*<0.05), indicating that IDO1 signaling limits T cell proliferation.

### Deficiency of IDO1 signaling promotes Th1 and CTL development in mice infected with *E. multilocularis*

**Figure 6:**
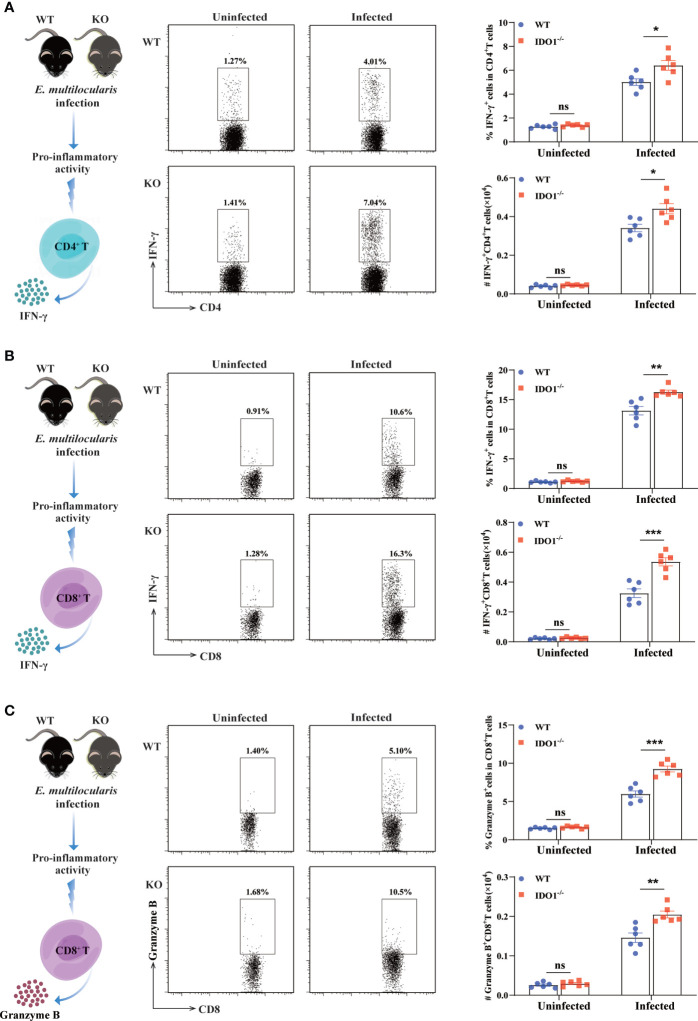
Enhanced Th1 and CTL development in IDO1^-/-^mice infected with *E multilocularis.* **(A, B)** Conceptual schematic diagram (left), representative flow cytometry plots (middle), and the frequency (upper right) and the absolute number (lower right) of IFN-γ secreting CD4^+^ T cells **(A)**, IFN-γ secreting CD8^+^ T cells **(B)**, and granzyme B secreting CD8^+^ T cells **(C)** of the IDO1^-/-^ and wild-type mice at 3 months post-infection with *E multilocularis* (n = 6/group). CD45^+^CD3^+^CD4^+^ and CD45^+^CD3^+^CD8^+^ cells were gated respectively for panel **(A)** and **(B, C)**. Data are pooled from 2 independent experiments, and presented as the mean ± SEM. ns, not significant; *, *p* < 0.05; **, *p*< 0.01; ***p< 0.001.

To further characterize the impact of IDO1 signaling on the phenotype of T cells during *E. multilocularis* infection, we evaluated the pro-inflammatory activity of CD4^+^- and CD8^+^- T cells by examining their secretions of IFN-γ and granzyme B using flow cytometry. In uninfected IDO1^-/-^ and WT mice, both CD4^+^- and CD8^+^- T cells displayed a lower percentage and absolute number of IFN-γ- and granzyme B- secreting cells. In contrast, the percentage and absolute number of IFN-γ-producing CD4^+^- and CD8^+^- T cells were significantly higher in infected IDO1^-/-^ mice compared to that in infected WT mice (*p*<0.001, <0.01, or <0.05, **Figures 6A, B**). Furthermore, a remarkably increased frequency of granzyme B-producing CD8^+^ T cells was observed in infected IDO1^-/-^ mice compared to that in infected WT mice (*p*<0.01 or <0.001, **Figure 6C**), indicating that IDO1 suppresses the cytotoxic function of CD8^+^ T cells during *E. multilocularis* infection.

In conclusion, these results demonstrate that IDO1 signaling play an important role in negatively regulating T cell immune responses by preventing DCs maturation in mice infected with *E. multilocularis*.

## Discussion

Immune tolerance is one of the major hallmarks of *E. multilocularis* infection (44). During the infection, various host immune cells interacted with the parasites primarily in the liver and formed a granulomatous inflammatory microenvironment, among which the pivotal role of DCs has long been appreciated (33, 36, 37). As previously reported, immune tolerance mediated by DCs was induced at the early stage of infection, which likely accounts for the generation of immunosuppressive microenvironment in the liver, leading to the immune evasion by the forementioned helminthic parasites (35). However, only few investigations have so far been carried out towards the identification and characterization of DCs functions during *E. multilocularis* infection.

DCs are professional antigen-presenting cells (APCs) with unique phenotypic markers and immunologic functions (45). Along with the maturation of DCs after antigen uptake, the expression of surface markers such as the MHC II and costimulatory molecules CD86 and CD80 are upregulated. Upon migration to the peripheral immune organs, mature DCs interact with naive T cells to promote T cell activation and differentiation (46). In this study, we evaluated the expression of MHC II, CD80 and CD86 on DCs in mice infected with *E. multilocularis*. Our data showed that the expression of the forementioned molecules on DCs was significantly increased on DCs in infected IDO1^-/-^ mice compared to that in infected wild-type mice, suggesting that IDO1 is a negative regulator of DCs maturation during *E. multilocularis* infection. In addition, chemokine receptors CXCR4 and CCR7 have been appreciated for the key roles on DC trafficking in chronic infectious diseases (47). In this investigation, we compared their expression on DCs in mice infected with *E. multilocularis* in the presence or absence of IDO1 signaling. We found that the expressions of CXCR4 and CCR7 was significantly enhanced on DCs in infected IDO1^-/-^ mice compared to infected wild-type mice. Taken together, our data are the first to identify that IDO1 plays an important role in *E. multilocularis* infection by repressing DCs maturation. However, the detailed mechanism of IDO^+^ DCs orchestrating the immune tolerance remains enigmatic, especially the effects of the immunomodulatory molecules secreted by the parasites.

Early studies have shown that there is a positive correlation between IFN-γ production and parasiticidal effects during infection of *E. multilocularis* (38, 48–50). In the present study, the frequency or the absolute number of the activated and proliferative CD4^+^- and CD8^+^- T cells were significantly higher in infected IDO1^-/-^ mice compared to that in infected wild-type group. More impressively, both CD4^+^- and CD8^+^- T cells secreted significantly higher IFN-γ in absence of IDO1 signaling, suggesting that IDO1 signaling negatively regulates the IFN-γ production comprehensively in major T cell subsets in *E. multilocularis*-infected mice. Moreover, the cytotoxic potential of CD8^+^ T cells, as represented by granzyme B expression, was significantly increased in infected IDO1^-/-^ mice compared to that in infected wild-type counterpart. Recent research findings have shown that overexpression of IDO1 in DCs facilitates T cell anergy in non-infectious disease models (22, 51). This effect occurs mainly through a mechanism called bystander suppression, by which the minor IDO1^+^ DCs population is able to suppress TCR signaling (52, 53). Interestingly, our data demonstrate that IDO1^+^ DCs not only inhibit the activation and clonal expansion of both CD4^+^- and CD8^+^- T cells, but also suppress their differentiation in context of *E. multilocularis* infection.

The microarray data has indicated IDO1 was significantly up-regulated in *E. multilocularis*-infected liver tissue (54). Accordingly, our data demonstrated that, in the spleen, IDO1 was highly upregulated in mice upon infection with *E. multilocularis*. We then identified IDO1 signaling as an important mediator in programming DCs to the tolerogenic state during *E*. *multilocularis* infection. Our data are first to demonstrate the essential role of IDO1 signaling in regulating immune responses *in vivo* during *E. multilocularis* infection. We also established the direct link between the impressive properties of IDO1 signaling and impaired maturation of DCs in spleens of mice infected with *E. multilocularis.* More importantly, compared to infected IDO1^-/-^ mice, infected wild-type mice exhibited higher volumes of metacestode tissue and higher parasite load as determined by MRI scanning and wet weight measurement. Indeed, immune tolerance is a hallmark of AE, and IDO1^+^ DCs are one of the key immune subsets to mediate this effect (55). Our results indicate that engagement of the IDO1 signaling promotes metacestode progression during infection with *E. multilocularis.*

In conclusion, we have described an essential role of IDO1 signaling on negatively regulating T cell responses by preventing DCs maturation in *E. multilocularis* infected mice. These data may contribute to an improved understanding of the immunoregulation mechanisms of IDO1 signaling underlying the *E. multilocularis* pathogenesis.

## Data availability statement

The original contributions presented in the study are included in the article/**Supplementary Material**. Further inquiries can be directed to the corresponding authors.

## Ethics statement

All animal experiments were performed in accordance with the guidelines of Institutional Animal Care and Use Committee of the Qinghai University Affiliated Hospital (P-SL-2022-031).

## Author contributions

HF, YF, and GL conceived the study. RM, YF, YZ, and YM conducted the experiments. RM, YF, and GL analyzed the data. RM, HF, GL, and YF wrote the paper. All authors contributed to the article and approved the submitted version.

## Funding

This work was financially supported by the National Natural Science Foundation of China (31960708), and the Applied Basic Research of Qinghai Province in China (2021-ZJ-724).

## Conflict of interest

The authors declare that the research was conducted in the absence of any commercial or financial relationships that could be construed as a potential conflict of interest.

## Publisher’s note

All claims expressed in this article are solely those of the authors and do not necessarily represent those of their affiliated organizations, or those of the publisher, the editors and the reviewers. Any product that may be evaluated in this article, or claim that may be made by its manufacturer, is not guaranteed or endorsed by the publisher.

## Supplementary material

Supplementary Figure 1 Expression of IDO1 on DCs in IDO1^-/-^ mice. **(A)**. Representative flow cytometry plots of IDO1 expression on CD11c^+^MHC II^+^ DCs in infected and non-infected IDO1^-/-^ and wild-type (WT) mice. **(B)**. The quantification of the frequency of IDO1 expressing CD11c^+^MHC II^+^ DCs (n = 5/group). CD45^+^MHC II^+^CD11c^+^ cells were gated. Data are presented as the mean ± SEM. FMO, full minus one staining control. ***, *p*<0.001; *****p*< 0.0001.

Supplementary Figure 2 IDO1 signaling limits DCs maturation in *E. multilocularis*-infected mice. **(A-F)**. Representative of the flow cytometry histogram of CD80, CD86, MHCII, CD11b, CXCR4, and CCR7 within splenic DCs of the IDO1^-/-^ and WT mice at 3 months post-infection with *E. multilocularis* (n = 6/group). CD45^+^MHC II^+^CD11c^+^ cells were gated. ns, not significant; *, *p<*0.05; **, *p*< 0.01; ***, *p*< 0.001. Abbreviations: PSCs, protoscoleces; p.i., post infection; i.i., injected intraperitoneally.

Supplementary Figure 3 IDO1^-/-^ DCs induce more robust T cell stimulatory effects *in vitro*. Splenic DCs were purified from *E. multilocularis* infected IDO1^-/-^ and WT mice, and co-cultivated with naïve splenic T cells for 4 days. T cell stimulatory effects of DCs were determined using the CFSE cell proliferation kit. **(A)**. Representative flow cytometry histograms of the CFSE dilution for T cells. **(B)**. The quantification of the CFSE dilution for T cells (n = 4/group). *, *p<*0.05; ***, *p*< 0.001.
