# Supplementary figures and images for: Indoleamine 2,3-dioxygenase 1 signaling orchestrates immune tolerance in Echinococcus multilocularis-infected mice
Source: Front Immunol. 2022 Nov 11;13:1032280. doi: 10.3389/fimmu.2022.1032280 (PMC9691980; doi:10.3389/fimmu.2022.1032280)

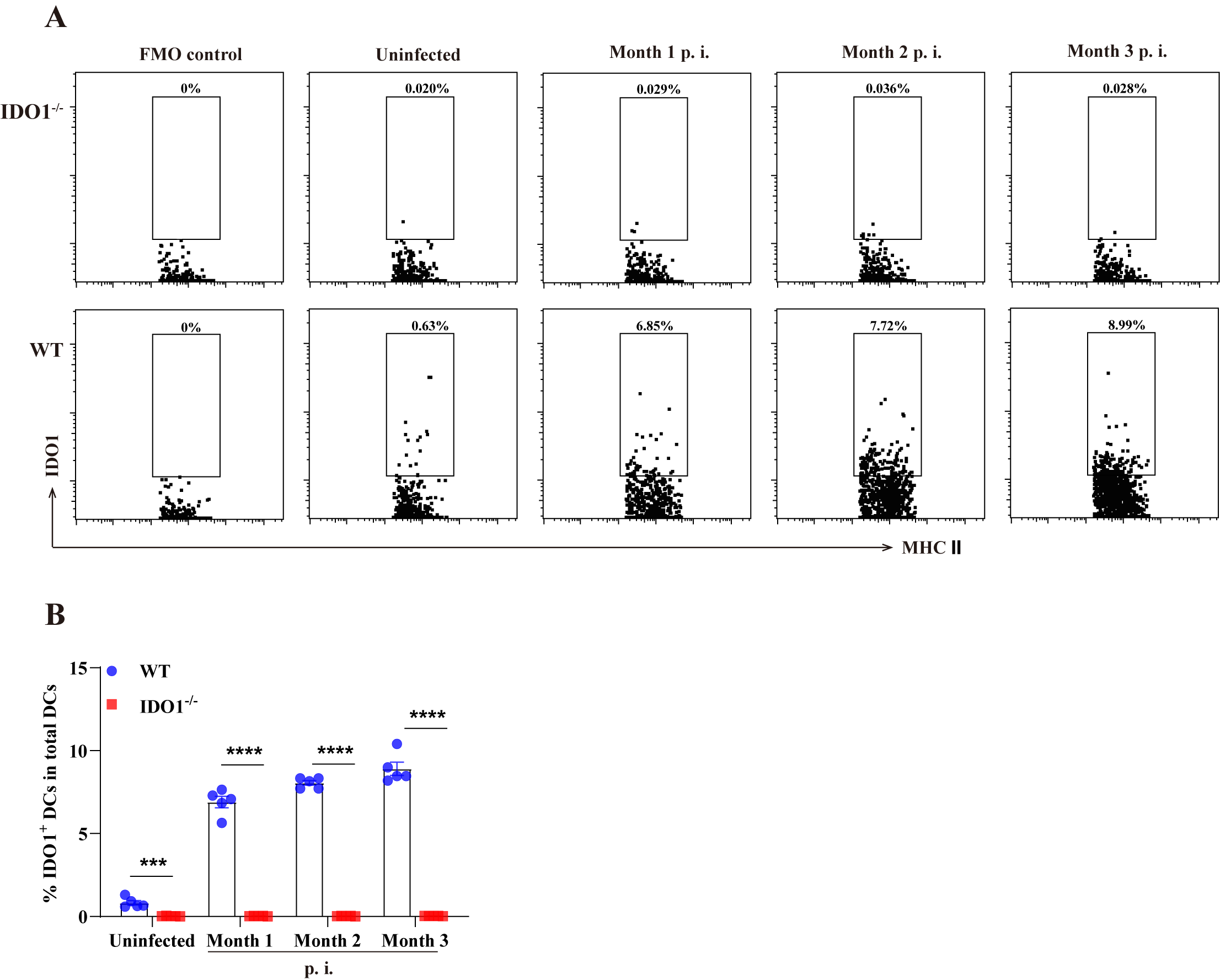

Supplement: Supplementary Figure 1 — Expression of IDO1 on DCs in IDO1-/- mice. (A). Representative flow cytometry plots of IDO1 expression on CD11c+MHC II+ DCs in infected and non-infected IDO1-/- and wild-type (WT) mice. (B). The quantification of the frequency of IDO1 expressing CD11c+MHC II+ DCs (n = 5/group). CD45+MHC II+CD11c+ cells were gated. Data are presented as the mean ± SEM. FMO, full minus one staining control. ***, p<0.001; ****p< 0.0001. [file Image_1.tif]

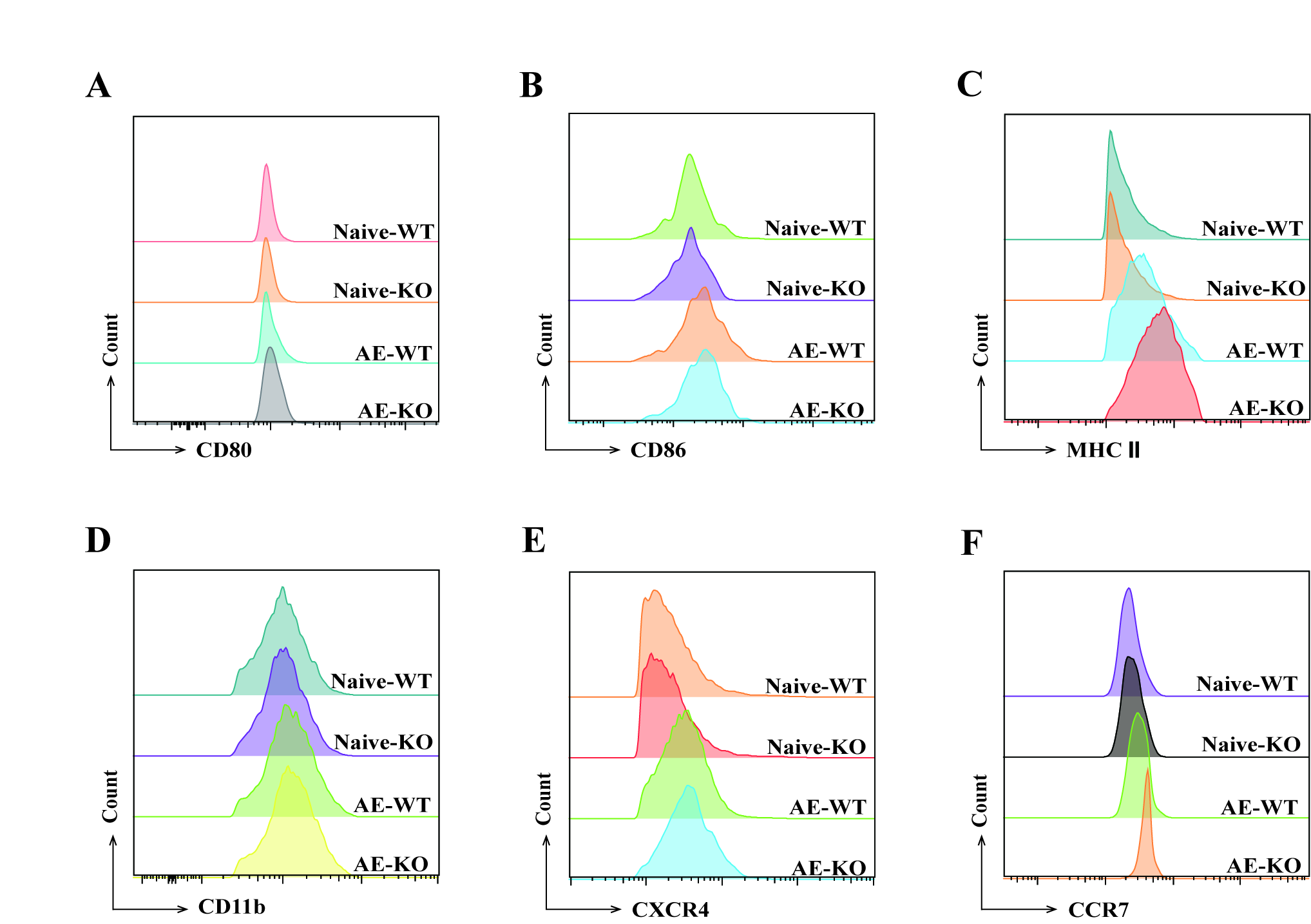

Supplement: Supplementary Figure 2 — IDO1 signaling limits DCs maturation in E. multilocularis-infected mice. (A-F). Representative of the flow cytometry histogram of CD80, CD86, MHCII, CD11b, CXCR4, and CCR7 within splenic DCs of the IDO1-/- and WT mice at 3 months post-infection with E. multilocularis (n = 6/group). CD45+MHC II+CD11c+ cells were gated. ns, not significant; *, p<0.05; **, p< 0.01; ***, p< 0.001. Abbreviations: PSCs, protoscoleces; p.i., post infection; i.i., injected intraperitoneally. [file Image_2.tif]

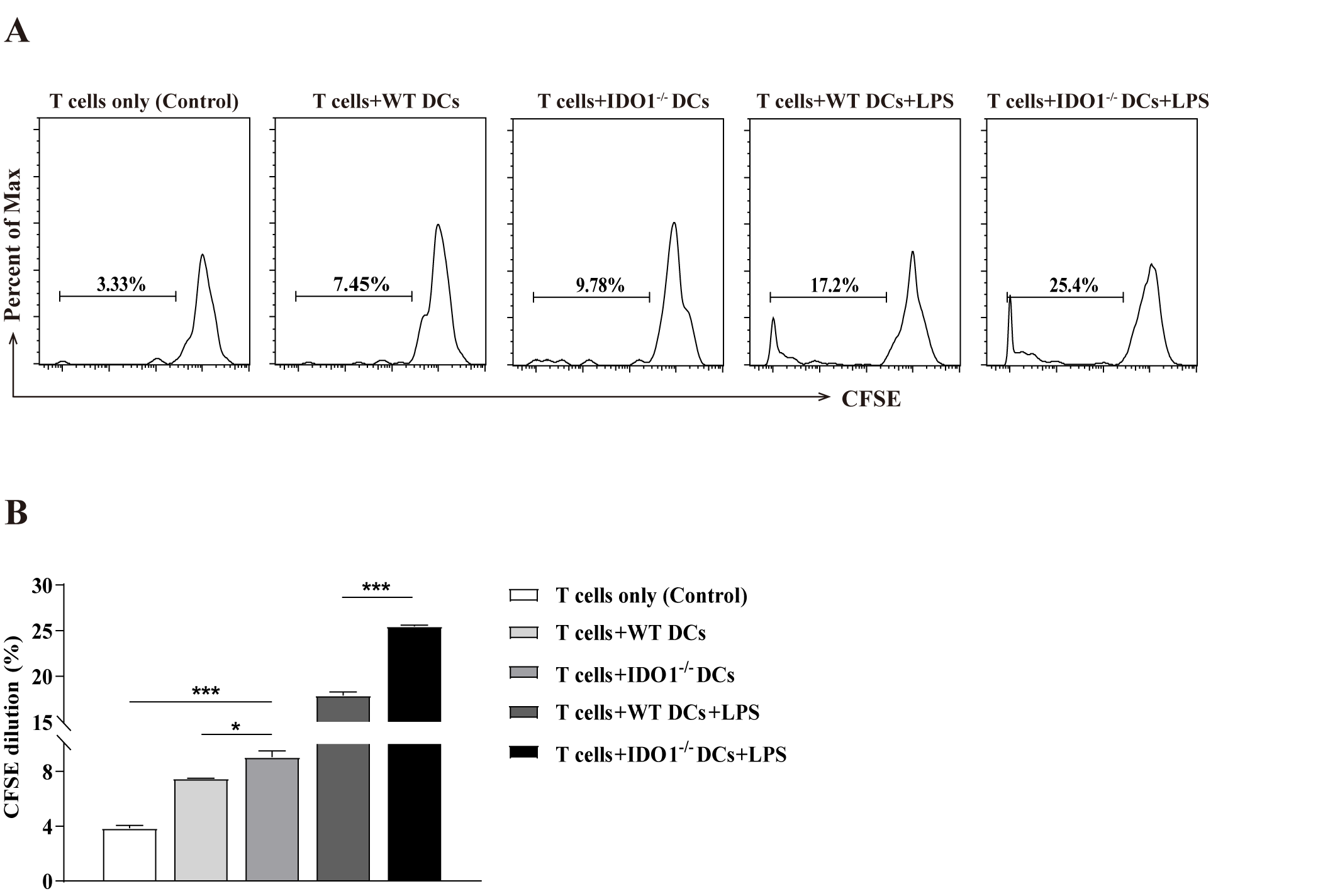

Supplement: Supplementary Figure 3 — IDO1-/- DCs induce more robust T cell stimulatory effects in vitro. Splenic DCs were purified from E. multilocularis infected IDO1-/- and WT mice, and co-cultivated with naïve splenic T cells for 4 days. T cell stimulatory effects of DCs were determined using the CFSE cell proliferation kit. (A). Representative flow cytometry histograms of the CFSE dilution for T cells. (B). The quantification of the CFSE dilution for T cells (n = 4/group). *, p<0.05; ***, p< 0.001. [file Image_3.tif]
